# Identification of a familial cleidocranial dysplasia with a novel *RUNX2* mutation and establishment of patient-derived induced pluripotent stem cells

**DOI:** 10.1007/s10266-021-00674-5

**Published:** 2021-11-15

**Authors:** Atsuko Hamada, Hanae Mukasa, Yuki Taguchi, Eri Akagi, Fumitaka Obayashi, Sachiko Yamasaki, Taku Kanda, Koichi Koizumi, Shigeaki Toratani, Tetsuji Okamoto

**Affiliations:** 1 Department of Molecular Oral Medicine and Maxillofacial Surgery, Division of Applied Life Science, Graduate Institute of Biomedical and Health Science, Hiroshima University, 1-2-3, Kasumi, Minami-ku, Hiroshima-city, Hiroshima 734-8553 Japan; 2 Department of Molecular Oral Medicine and Maxillofacial Surgery, Graduate School of Biomedical and Health Science, Hiroshima University, Hiroshima, Japan; 3 School of Medical Sciences, The University of East Asia, Shimonoseki, Yamaguchi 751-8503 Japan; 4 Present Address: Mukasa Dental Clinic, Kanagawa, Japan

**Keywords:** RUNX2, Mutation, Dysplasia, Cleidocranial, iPSC

## Abstract

Cleidocranial dysplasia (CCD) is an autosomal dominant hereditary disease associated with the gene *RUNX2*. Disease-specific induced pluripotent stem cells (iPSCs) have emerged as a useful resource to further study human hereditary diseases such as CCD. In this study, we identified a novel CCD-specific *RUNX2* mutation and established iPSCs with this mutation. Biopsies were obtained from familial CCD patients and mutation analyses were performed through Sanger sequencing and next generation sequencing. CCD-specific human iPSCs (CCD-hiPSCs) were established and maintained under completely defined serum, feeder, and integration-free condition using a non-integrating replication-defective Sendai virus vector. We identified the novel mutation *RUNX2_c.371C*>*G* and successfully established CCD-hiPSCs. The CCD-hiPSCs inherited the same mutation, possessed pluripotency, and showed the ability to differentiate the three germ layers. We concluded that *RUNX2_c.371C*>*G* was likely pathogenic because our results, derived from next generation sequencing, are supported by actual clinical evidence, familial tracing, and genetic data. Thus, we concluded that hiPSCs with a novel CCD-specific *RUNX2* mutation are viable as a resource for future studies on CCD.

## Introduction

Cleidocranial dysplasia (CCD [MIM 119600]) was first described as cleidocranial dysostosis by Marie and Sainton [1]. The incidence of CCD is estimated to be one per one million people. While familial cases of CCD are inherited in an autosomal dominant manner with marked phenotypic variability, many cases of this disorder appear to be sporadic [2]. CCD is characterized by skeletal anomalies such as delayed closure of the cranial sutures, hypoplastic clavicles, short stature, and multiple dental abnormalities, including delayed eruption of permanent teeth and existence of supernumerary teeth. The delay of permanent teeth replacement or lack of eruption of permanent dentition leads to a lack of vertical growth of the maxilla, resulting in horizontal overgrowth of the mandible [3]. To obtain normal occlusion, dental management of CCD patients should be initiated under early diagnosis [4].

The gene associated with CCD is *runt-related gene 2 (RUNX2)* (previously called Cbfa1) [5–7], which is located on chromosome 6 (6p21). RUNX2 is a transcription factor that belongs to the RUNX family, which consists of RUNX1, RUNX2, and RUNX3. RUNX2 induces the proliferation of suture mesenchymal cells and their commitment to osteoblast-lineage cells by increasing the expression of *hedgehog*, *FGF*, *WNT*, and *PTCH* signaling genes [8]. In chondrocytes, RUNX2 acts on the differentiation of pre-hypertrophic chondrocytes into hypertrophic chondrocytes [9–12]. The runt homology domain (RHD) of RUNX2, is composed of 128 amino acids and is responsible for heterodimerization with Cbfb, a cofactor that enhances the DNA affinity of the subunit [13, 14]. A nine-amino acid sequence (PRRHRQKLD) located behind the RHD, behaves as a nuclear localization signal (NLS) [15]. Both the RHD and the NLS have been conserved in all RUNX proteins across the species. This evolutionary conservation of the RHD and NLS indicates that most missense mutations involving the runt domain are likely to affect protein function and lead to haploinsufficiency [16]. In CCD patients, many monoallelic mutations of *RUNX2* have been identified, including deletion, missense, nonsense, and frameshift mutations [2, 17, 18]. Most of these mutations are clustered in the N-terminal RHD with several positions emerging as mutational hotspots [2]. Of these, arginine residues are thought to be particularly prone to mutagenic events through CpG-directed methylation [16]. The missense mutation RUNX2_R225Q, which has been reported in multiple cases worldwide, is possibly the most frequently found mutation in CCD [19–21]. The use of CCD disease-specific human induced pluripotent stem cells (CCD-hiPSCs) with RUNX2_R225Q in disease modeling has also been previously reported by us [22]. With the aim of further understanding human hereditary diseases, disease-specific iPSCs were first established by Okada et al. [23]. Examples of iPSC use include the modeling of chondrodysplasias in vitro and drug dispositioning using disease-specific iPSCs [24]. In this study, we identified the novel mutation *RUNX2*_c.371C>G in familial CCD patients and established CCD-specific hiPSCs bearing this novel *RUNX2* mutation.

## Material and methods

### Patient information

**Fig. 1:**
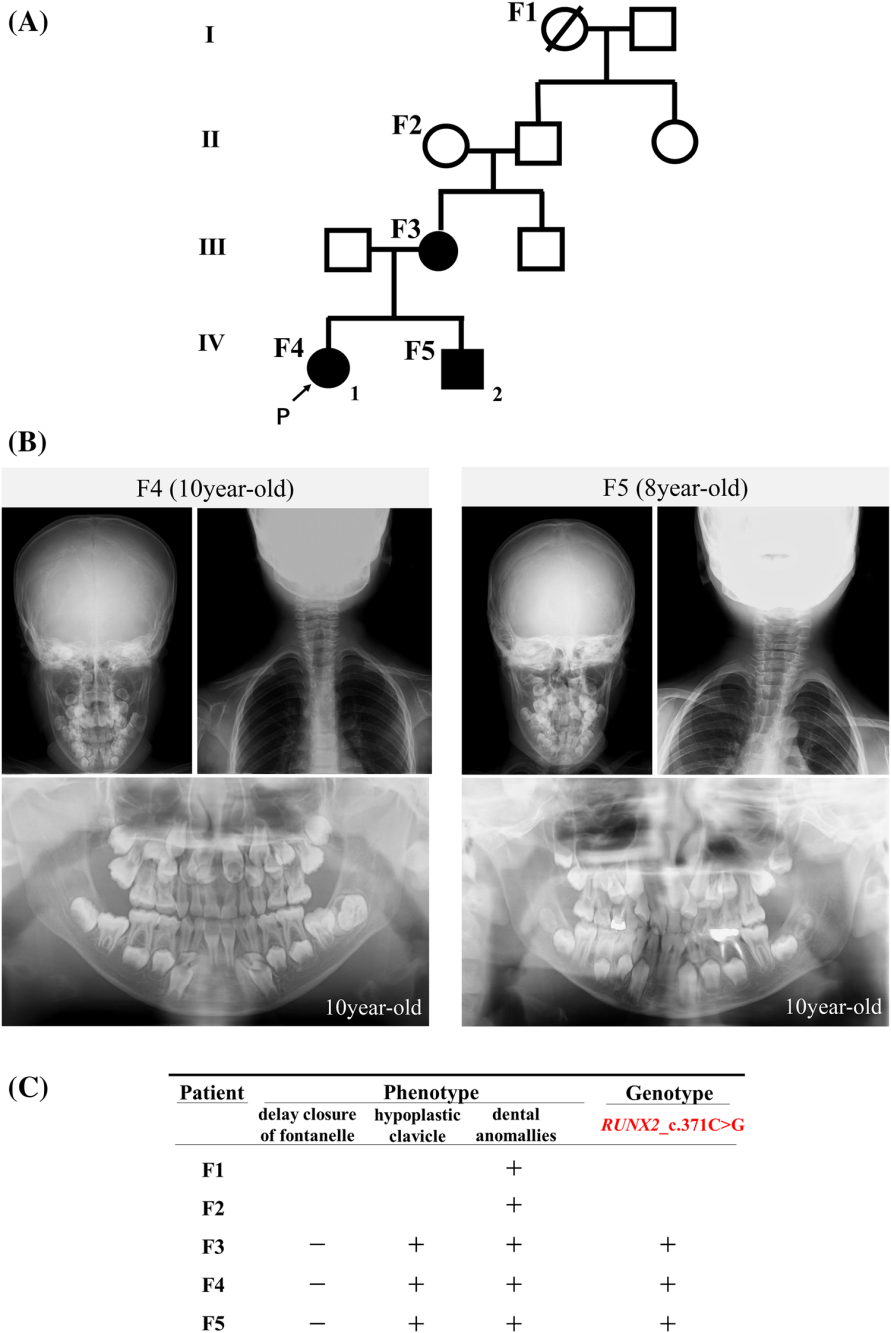
Pedigree and phenotype of familial Cleidocranial dysplasia (CCD). **A** Pedigree of familial CCD was shown. **B** Cephalometric radiogram and panotamic X-ray photos of F4 and F5. (C) F3, F4, and F5 shared phenotypes and genotype. The phenotype of cranial and clavicle both in F1 and F2 were not detected because of difficulties in visiting our hospital

The patient (F4) (Fig. 1A), an 8-year-old girl, visited pediatric dentistry in our hospital with the main complaint of malocclusion. According her medical history, she had been clinically diagnosed with CCD by her pediatrician during infancy. She was referred to our department at 10-year-old and was diagnosed with CCD according to criteria, which included delayed closure of the cranial sutures, presence of supernumerary teeth, and non-eruption of permanent teeth (Fig. 1B). The patient underwent extraction of supernumerary teeth and odontoma, pulling of unpacked permanent teeth, and orthognathic surgery under general anesthesia. The patient's younger brother (F5) (Fig. 1A) was also clinically diagnosed with CCD and introduced to our department because of malocclusion. He underwent extraction of supernumerary teeth and pulling of unpacked permanent teeth under local anesthesia. The patient's mother (F3) (Fig. 1A) had started orthodontic treatment in our hospital a few years ago and was clinically diagnosed with CCD (Fig. 1B). She had undergone several tooth extractions in another hospital before her first visit to our hospital making the details of her treatment unavailable. The patient's grandfather (F2) (Fig. 1A) seemed to exhibit a skeletal mandibular protrusion, and her great-grandmother (F1) (Fig. 1A) seemed to have several supernumerary teeth. Although they (F1 and F2) were not diagnosed in our hospital, they were most likely suffering from CCD. The pedigree and phenotype are shown in Fig. 1A and 1C.

### Mutation analysis with Sanger sequencing and next generation sequencing (NGS)

The patient’s genomic DNA was isolated from blood samples, dental pulp cells (DPCs) and CCD-DPC-hiPSCs with a QIAamp® DNA mini kit (Qiagen, Valencia, CA) according to the manufacturer’s protocol. To identify *RUNX2* mutations, Sanger sequencing was performed with the primers we described previously [22]. The PCR product was purified using a Wizard® SV Gel and PCR Clean-Up System (Promega, Madison, WI) and sequenced directly with a CEQ8000 Beckman system (Beckman-Coulter, Brea, CA).

To further search for pathogenic mutations in genes other than *RUNX2* in this CCD family, targeted resequencing was performed with a MiSeq using a TruSight one panel (Illumina, San Diego, CA, USA), which is designed to comprehensively cover more than 4800 genes involved in diseases. Sequencing was done according to the manufacturer’s instructions using DNA derived from blood samples and dental pulp samples from F3 and F5. Data analysis was performed using the Illumina VariantStudio 3.0.

### Isolation of dental pulp cells (DPCs) and induction of CCD-DPC-hiPSCs

Isolation of DPCs and induction of DPC-hiPSCs were performed as described previously [25]. Briefly, DPCs were cultured in RD6F serum-free medium [26] for 14 days at 37 °C in a humidified atmosphere of 95% air/5% CO_2_. Then, DPCs at a density of 1 × 10^5^ cells were infected with SeVdp (KOSM) [27], which does not integrate into the host genome, at a multiplicity of infection of 6 in a gelatin (Millipore, Billerica, MA)-coated 12-well plate. After overnight culture the infected cells were trypsinized, seeded at a density of 1.0 × 10^4^ cells/well onto a fibronectin (2 μg/cm^2^) (Sigma-Aldrich, St. Louis, MO)-coated 6-well plate with serum-free hESF9 medium [28–30], and incubated at 38 °C under the conditions described above. Approximately 14 days after infection, human embryonic stem cell (ESC)-like colonies were mechanically picked, and subsequently expanded on fibronectin-coated dishes in hESF9 with TGF-β1 (2 ng/mL) or activin A (10 ng/mL) at 37 °C in 95% air/5% CO_2_ as reported previously [31].

### Characterization of CCD-DPC-hiPSCs in vitro

The hiPSCs were characterized as described previously [25, 32]. Briefly, to confirm the pluripotency of the CCD-DPC-hiPSCs, reverse transcription-polymerase chain reaction (RT-PCR) of pluripotent markers (*OCT, NANOG, SOX2, and REX1*) was performed using OnePCR™ (GeneDireX, Inc. Taoyuan, Taiwan). Next, to determine the in vitro differentiation capacity of the CCD-DPC-hiPSCs, we performed an embryoid body (EB) assay. Differentiated cells were fixed, stained with antibodies (against β-III tubulin, MAP, α-smooth muscle actin, and α-fetoprotein), and visualized under a Zeiss inverted LSM 700 confocal microscope (Carl Zeiss GmbH, Jena, Germany).

### Chondrocyte induction under completely serum-free conditions

The hiPSCs were detached with TrypLE/ EDTA, and 1 × 10^5^ cells per well were in seeded in a low attachment U bottom prime surface 96-well plate in hESF6 without FGF2 and heparin and cultured for 7 days to make EBs. Then, 5–7 EBs were transferred to a gelatin-coated 35 mm dish. After approximately 7 days, mesenchymal stem-like cells had grown out from the EBs and spread to confluence in the dish. To remove the EB mass, the detached cells were filtered through a 70 µl filter. After several passages through the filter using hESF9 with FGF2 and heparin, the cells were analyzed with FACS (Sony, cell sorter, Tokyo, Japan) using anti-MSC marker (CD73, CD90, CD105) antibodies (Miltenyi Biotec) on 4th week. The induced MSCs were seeded on a fibronectin-coated 60 mm dish at a concentration of 1.0 × 10^6^ cells per dish with hESF9. Chondrogenic medium consisted of hESF6f as a base medium supplemented with Wnt3A (10 ng/ml) (R&D, Minneapolis, MN) and activin (10 ng/ml) (R&D) for 4.5–4.7 weeks and then BMP2 (50 μg/ml) (R&D), TGFβ1 (10 ng/ml) (R&D), GDF5 (10 ng/ml) (Origene MD, USA), and FGF2 (10 ng/ml) (R&D) for 4.7–10 weeks. On the 6th week, the cells were detached in a sheet using a p-1000 pipet tip, transferred to a 15 ml tube, centrifuged at 200×*g* for 5 min (KUBOTA corporation, Tokyo, Japan), and kept in an incubator at 37 °C in a humid atmosphere of 95% air/5% CO_2_. The tube was centrifuged at 7×*g* for 5 min each day over 6–10 weeks. This protocol was a modified version of the process for chondrogenic differentiation of hiPSCs reported by Yamashita et al. [33]. At 10th week, cartilages were implanted in the dorsal flank of SCID (CB17/Icr-Prkdcscid/CrlCrlj) mice for more 4 weeks. After fixation with 4% PFA and decalcification with ethylenediaminetetraacetic acid, each slice was stained with Alcian blue/PAS and Safranin O.

## Results

**Fig. 2:**
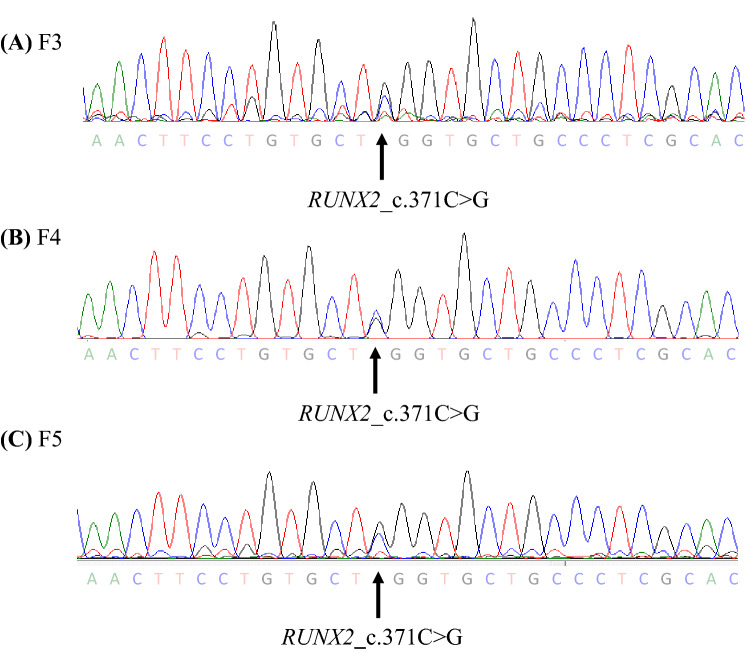
Direct sequencing of *RUNX2* in familial Cleidocranial dysplasia (CCD). The results of direct sequencing of *RUNX2* exon 3 are shown. The *RUNX2*_c.371C>G were detected in F3–F5 (**A**–**C**)

Using Sanger sequencing, *RUNX2_c.371C*>*G* was detected in individuals F3, F4, and F5 (Fig. 2). To screen for other pathogenic mutations affecting CCD, we performed NGS on an MiSeq using a TruSight one panel for familial CCD and detected three shared mutations, including, *RUNX2_c.371C*>*G*, *SIL1_c.617T*>*A*, and *SOX9_c.724A*>*C*. *SIL1* is the gene responsible for autosomal recessive Marinesco-Sjögren syndrome (MIM 608005). Though *SIL1_c.617T*>*A* was predicted to be ‘disease-causing’ in silico, the detected mutation was heterozygous, indicating non-pathogenicity. SOX9, the Sox family of transcriptional regulators, is known to function during chondrocyte differentiation, and its deficiency leads to skeletal malformation [34]. However, the detected mutation, SOX9_c.724A>C_p.K242Q, was not located in the DNA binding domain. Thus, we eliminated the *SIL1_c.617T*>*A* and *SOX9_c.724A*>*C* mutations from the group of mutations considered pathogenic. The base substitution ‘c.371C>G’ in *RUNX2,* which was predicted to be ‘disease-causing’ by Mutation Taster, ‘probably damaged’ by PolyPhen-2, and ‘deleterious’ by the SIFT software*,* indicates a substitution of serine to tryptophan at codon 124, p.S124W, which is in the RHD. Based on this information, we predicted that RUNX2_c.371C>G_p.S124W was a ‘likely pathogenic’ mutation for CCD. However, RUNX2_c.371C>G_p.S124W was not found in Togo Var or Exome Variant Server, whereas in dbSNP and Clinvar, this mutation was submitted as NM_001024630.4(RUNX2):c.371C>G (p.Ser124Trp) in January 2021 and classified as a variant of uncertain significance (VUS). However, as far as we have investigated, clinical case of CCD with this mutation has not been reported anywhere.

**Fig. 3:**
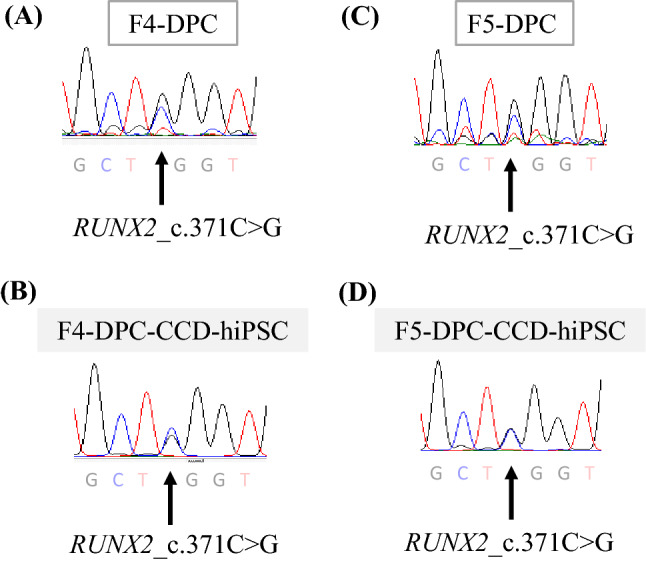
Direct sequencing of *RUNX2* in dental pulp cells (DPCs) and DPC-CCD-hiPSCs. The mutation in DPC (**A** and **C**) were hereditary in DPC-CCD-hiPSC (**B** and **D**) respectively

**Fig. 4:**
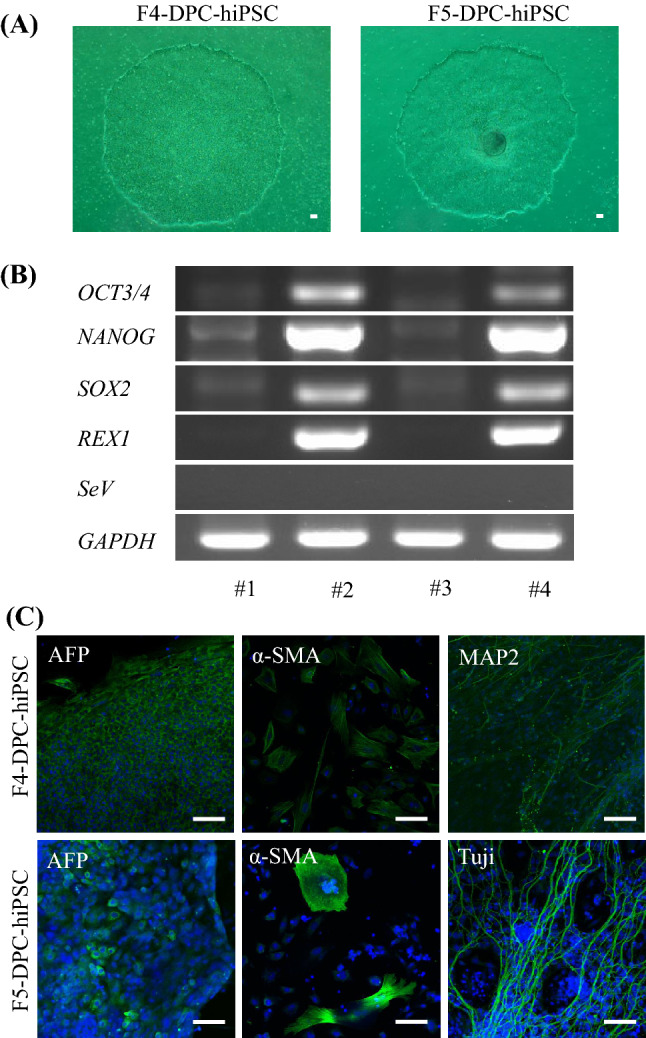
Characterization of DPC-CCD-hiPSCs. **A** Phase contrast images of DPC-CCD-hiPSCs (F4-DPC-iPS; clone 6 at passage 21, F5-DPC-iPS; clone 3 at passage 21). **B** Gene expression of pluripotent markers by RT-PCR (#1; F4-DPC, #2; F4-DPC-CCD-iPSC, #3; F5-DPC, #4; F5-DPC-CCD-iPSC). Although *NANOG* and *SOX2* were weakly detected before reprogramming, *OCT3/4*, *NANOG*, *SOX2*, and *REX1* were strongly expressed after reprogramming. SeVdp was not detected under any conditions. **C** Immunofluorescence staining of differentiation markers in DPC-CCD-hiPSCs after 3 week of differentiation in vivo (β-III tubulin, MAP2, smooth muscle actin (SMA), and alpha fetoprotein (AFP)). Each bar indicates 100 μm in length

CCD-derived disease-specific hiPSCs inherited the RUNX2 mutation c.371C>G (Fig. 3), exhibiting ESC-like colonies in phase contrast images (Fig. 4A). The cells were positive for *OCT*, *NANOG*, *SOX2*, and *REX1* in PCR analyses (Fig. 4B). In vitro, these cells differentiated into three germ layers: the endoderm, mesoderm, and ectoderm (Fig. 4C). Together, these properties demonstrate that the cells were pluripotent and had the ability to differentiate.

## Discussion

The algorithms of SIFT, PolyPhen-2, and Align-GVGD, etc. were recently developed to predict the effects of missense changes on protein structures [35]. According to the algorithms, NM_001024630.4(RUNX2): c.371C>G (p.Ser124Trp) was predicted ‘likely pathogenic’ because the serine residue was moderately conserved and there was a large physicochemical difference between serine and tryptophan. Since Yoshida et. al. reported that the mutations (151 fs, R179X, R176W, K204N, T206I, and R211W) in RHD domain showed no DNA binding [2], we predicted the detected mutation c.371C>G_p.S124W, which located in RHD domain, also lacks DNA binding ability. As this variant had not been reported in individuals with CCD-associated phenotypes in the literature, it was classified as a VUS. In this report, we identify, for the first time, the RUNX2 mutation c.371C>G_p.S124W in a CCD family.

The CCD family reported in this study exhibited the common phenotypes of open suture, hypoplastic clavicles, and supernumerary teeth. Although the patients showed no delay in fontanelle closure, in general, our results matched those of a previous study [2]. Short stature was only observed in female patients (F3 and F4). This finding contradicts those of the animal model report by Choi et al., in which male C57BL/6 mice heterozygous for Runx2 with a C-terminal deletion exhibited a significantly lower growth than wild type mice, while the equivalent female heterozygotes showed normal weight [36].

Patients F4 and F5 had 8 and 10 supernumerary teeth, respectively. Previous studies have reported that in patients with CCD, the number of supernumerary teeth ranged from 0 to 21, and that these teeth were frequently found as maxillary incisors and mandibular premolars [37, 38]. Understanding the relationship between the mutations in RUNX2 and CCD-associated phenotypes, including the final height of patients or the number of supernumerary teeth, may enable the early initiation of effective treatments, such as therapy with recombinant human growth hormone at an appropriate stage. In previous study, we reported that the cartilage tissues derived from 3-dimentional cultured-CCD-hiPSCs, which established from DPC of CCD patient (RUNX2_R225Q), indicated lack of cartilaginous elements [22]. Furthermore, after 4 weeks inoculation of cartilage derived from CCD-hiPSCs in dorsal flank of SCID (CB17/Icr-Prkdcscid/CrlCrlj) mice, the cartilage exhibited delayed ossification compared from those of control (Supplementary Figure). These findings might suggest that the mutation of the RUNX2 in the CCD inhibits proliferation and differentiation of hypertrophic chondrocytes or osteoblast-lineage indicating that the CCD-hiPSCs model could mimic some part of CCD pathology. On the other hand, it has been reported that regenerative medicine using anti-uterine sensitization-associated gene-1, which exerts an antagonistic effect against RUNX2 during tooth development, could control the tooth number [39, 40]. Using CCD-iPSC-derived cell types, we will be able to identify specific roles for RUNX2 in chondrocyte development, reveal disease occurring, and identify new therapeutic targets that would complement strategies to modulate the signaling properties of the RUNX2.

In conclusion, we report a novel mutation, RUNX2_c.371C>G_p.S124W, in Japanese familial CCD. Based on the clinical evidence and preliminary data mining using online tools and software, RUNX2_c.371C>G_p.S124W is a ‘likely pathogenic’ mutation for CCD. Based on previous findings and those of the current study, analyses of *RUNX2* mutations using CCD-derived iPS cell lines may contribute to the development of regenerative therapies for CCD.
